# Co-creation and prototyping of an intervention focusing on health literacy in management of malaria at community-level in Ghana

**DOI:** 10.1186/s40900-021-00302-0

**Published:** 2021-08-05

**Authors:** Millicent Addai Boateng, Eter Agyei-Baffour, Sanne Angel, Ofeibea Asare, Benjamin Prempeh, Ulrika Enemark

**Affiliations:** 1 Department of Public Health, Faculty of Health, Aarhus University, Aarhus, Denmark; 2 Department of Health Policy, Management and Economics, School of Public Health, College of Health Sciences, Kwame Nkrumah University of Science and Technology, Kumasi, Ghana; 3 Career Development Centre, College of Health Sciences, Kwame Nkrumah University of Science and Technology, Kumasi, Ghana; 4 Department of Communication Design, College of Art and Built Environment, Kwame Nkrumah University of Science and Technology, Kumasi, Ghana

**Keywords:** Health literacy, Co-creation, Board game, Intervention, Caregivers, Malaria, Ghana, West Africa

## Abstract

**Introduction:**

Collaborating with end-users to develop interventions tailored to fit unique circumstances is proposed as a way to improve relevance and effectiveness of an intervention. This study used a local needs driven approach to develop a health literacy intervention for caregivers in Ghana concerning management of malaria in children under 5 years.

**Method:**

For the period, November 2017–February 2019**,** we carried out the study using a three-phase framework including: 1) Needs assessment based on data from questionnaires, focus groups, individual interviews and observations, 2) Co-creation of a board game and brochures for health education at Child Welfare Clinics to address needs in health literacy concerning malaria and 3) Development of a prototype of the game, brochures as well as determining feasibility. In addition to the research team, health administrators, community health workers, designers and caregivers contributed to the development of the intervention.

**Findings:**

The needs assessment contributed to the development of interactive and useful materials including a board game and brochures, to help bridge the gaps in health literacy among caregivers. Co-creation of the materials and prototyping yielded a varying sense of ownership among stakeholders. End-users’ engagement and participation in developing the intervention resulted in a high interest and adherence to interventions. However, high attrition rates of health workers and caregivers’ inconsistent use of the Child Welfare Clinics challenged sustainability of this intervention.

**Conclusion:**

Co-creation led to an interactive intervention. The interactive nature of the board game and brochures resulted in a better caregiver-health provider relationship and a sense of recognition of a more participatory approach to health delivery. We recommend co-creation as an approach to develop needs-driven interventions in a context like Ghana. Still, a stronger buy-in at the top-level of health management would improve sustainability and reach a larger audience.

**Supplementary Information:**

The online version contains supplementary material available at 10.1186/s40900-021-00302-0.

## Plain English summary

**Introduction:**

To develop useful interventions, it is important to involve the users of the intervention in its development. Such interventions address the unique needs of the users. This study reports on the design and evaluation of an intervention which was designed to meet the health literacy needs of caregivers with children under 5 years in the management of malaria in Ghana.

**Method:**

The development process involved three phases. Firstly, the study identified the health literacy needs of caregivers using questionnaires, focus group discussions, interviews and observations. Secondly, there was collaboration with stakeholders in the design of a board game and brochures as health education tools to be used at the Child Welfare Clinics and lastly, the practicality of the designed materials as health education tools for caregivers was assessed.

**Findings:**

The intervention addressed the identified health literacy needs of caregivers. Stakeholders involved in the development process expressed ownership in varying degrees depending on the extent of their involvement. Users of the board game and brochures showed high interest and participation in the intervention delivery. Sustainability was challenged with periodic transfer and loss of health workers to career development, as well as irregular visits by some caregivers to the Child Welfare Clinics.

**Conclusion:**

The use of interactive game and brochures at the Child Welfare Clinics improved caregiver and health worker relationship and encouraged a participatory method of healthcare. For long-term sustainability and scale-out of the intervention, there is the need for more support of top-level management of health in the country.

**Supplementary Information:**

The online version contains supplementary material available at 10.1186/s40900-021-00302-0.

## Introduction

This paper describes the development of a community-based intervention in Ghana, a low-income setting, focusing on health literacy to increase knowledge in malaria prevention and early treatment practices among mothers with children under 5 years.

In 2018, about 93% of the estimated 228 million malaria cases worldwide were traceable to Africa [1]. In the same year, malaria in Ghana accounted for 4% of the global disease burden and 7% of the malaria disease burden in West Africa [2]. In 2019, the Ghana Malaria Indicator survey reported that 1 out of 7 children under 5 years tested positive to the disease [3] and the unfavourable clinical outcomes resulting from complicated malaria makes the disease one of great concern [4]. Although there has been a reduction in the percentage of deaths due to malaria in children under 5 years old from 15% in 2010 to 11% in 2016 [2], Ghana still battles with eradication of malaria as it ranks first among the top 10 causes of death [5]. One barrier to eradicate malaria is the misunderstanding of information, which hinders use of preventive measures and appropriate response to the disease [6]. This calls for attention to access the right health information and understand health information as important for early treatment and prevention of malaria.

When working with knowledge transfer, the ability of people to make informed health decisions is central to promote good health. The World Health Organisation (WHO) defines health literacy as the cognitive and social skills which determine the motivation and ability of an individual to gain access to understand and use information to promote and maintain good health [7]. Low levels of health literacy are associated with poor patient-provider communication, medicine adherence, treatment, self-management, use of healthcare and information and low uptake of preventive measures [8]. Okan et al. recommended that tailored health literacy interventions should be systematically developed and described in detail for possible replication in similar contexts to meet the needs of people with similar backgrounds [8].

The aim of this study was to develop and pilot a relevant intervention, as much as possible building on existing community programmes, to meet the practical needs of caregivers of children under 5 years in relation to health literacy to inform prevention and early treatment of malaria in children under 5 years in Ghana.

There are several approaches to developing public health interventions [6, 9–11], though with considerable overlap in the actions required [10]. Two recommended approaches include theory driven and evidence based approach. This study is both theory driven and evidence based, specifically using, the intervention mapping approach [10] as was applied to the OPtimal HEalth LIterAcy (Ophelia) protocol [12]. The Ophelia protocol is a protocol for health literacy profiling and engaging of a community to create and implement a health reform [12] . The Ophelia protocol builds on needs assessment, which is in line with the first step in the intervention mapping and further draws on the concept of co-creation in the development of a health literacy intervention [13]. The Ophelia protocol recommends a co-creation approach for development of solutions that meet identified needs to achieve positive health and equity outcomes for clients as well as ownership of the intervention [12] [13, 14]. Co-creation is here defined as the collaboration between academics or researchers and society in the development of knowledge or a product useful to the society [11, 15]. Involvement of users of the intervention promotes acceptability and feasibility [11, 15]. Thus, this approach as opposed to a one size fits all intervention, is likely to increase effectiveness [10]. It increases satisfaction, participation, ownership and sustainability towards positive societal changes on the part of users [10].

In addition to needs assessment and co-creation, this study adds the concept of prototyping as the third phase based on the framework of Hawkins et al. for public health interventions, which involves creation of materials or products [16]. This third phase uses prototyping to assess the feasibility of draft materials or products from an intervention prior to piloting and evaluation [16]. In summary, this study used a three-phase intervention mapping approach, a needs assessment, co-creation and prototyping to produce feasible materials acceptable to improve health literacy of mothers with children under 5 years in the prevention and early treatment of malaria.

Public health interventions are used in several ways to meet the needs of the target group of beneficiaries. In recent times, the use of games in health promotion interventions is catching up with other intervention methods [17–19]. Board games specifically, aim to affect lifestyle by passing on knowledge and its use could effectively change behaviours [17]. In this study, we build on the snakes and ladders board game, with inspiration from its use as health education tools in other studies [17, 20–24].

### The intervention

We designed a community-tailored Malaria Health Literacy intervention to address three health literacy dimensions using interactive materials consisting of a board game and brochures. The board game is played by mothers in groups of two to four and both game play and brochure discussions are facilitated by community health nurses at the child welfare clinics. Basically, it involves the use of malaria brochures, a snakes and ladders board game (mosquitoes and ladders), with supporting information on malaria as well as breastfeeding, complementary feeding and the hospital referral system. The intervention was intended to address the health literacy needs of mothers in three health literacy dimensions: having health provider support, navigating health system and understanding health information; respondents had low average mean scores in these three dimensions in the needs assessment. The design of this intervention sought to meet the health literacy needs of mothers using the socio-cultural strength of the society.

## Methods

### Study site

The study was carried out in two peri-urban districts in the Ashanti region of Ghana (Ejisu-Juaben and Kwabre East). Both are farming communities where most women are petty traders of farm products.

### Study design

In accordance with Hawkins et al. (2017) [16], the method used in the design of this intervention is described in three phases: 1. Needs assessment; 2. Co-creation (Co-production is used often in this study to emphasize production of the materials); 3. Prototyping [16]. The three-phase approach was based on evidence from scientific literature and primary data as well as knowledge of the key stakeholders and their expertise.

In line with the co-creation approach, stakeholders were introduced to the process after the needs assessment to deliberate on the relevance of the intervention concept. The key stakeholders included the Ejisu District Health Directorate (Ghana Health Service, GHS), the unit of health resource and learning materials (GHS), community health workers (GHS), community-based agents, a communication design researcher, public health researchers and ultimately mothers with children under 5 years who were also the intended beneficiaries of the intervention.

### Phase 1 needs assessment

The needs assessment comprised of both quantitative and qualitative elements with an aim to identify few health literacy dimensions where the need was most important and how to design and roll out the intervention.

#### Baseline survey

The aim of this cross-sectional survey was to assess health literacy needs and knowledge of caregivers on management of malaria in children under 5 years. Two questionnaires were administered to 1234 caregivers with children under 5 years, selected through multi-stage random sampling. Details on sampling is reported in another paper [25]. One questionnaire included questions to assess health literacy levels generally and the other questions to assess knowledge and practices of management of malaria based on the existing malaria community programmes.

Health literacy was measured using the Asante-Twi version of the Health Literacy Questionnaire (HLQ) [25]. The Health Literacy Questionnaire is a multi-dimensional tool designed to provide practitioners, organisations and governments with data on health literacy strengths and limitations at individual and population level [7, 12, 26]. The tool has been used in various groups of people ranging from the general population to policy makers, which makes it applicable to caregivers in this study [12, 26]. The tool stands out for its excellent psychometric properties, construct validity and strong reliability with an unbiased mean estimate of group differences [26].

The HLQ is a 44-item questionnaire using a Likert scale score [26] to classify respondents’ health literacy on nine dimensions (scales):

1. Feeling understood and supported by healthcare providers
2. Having sufficient information to manage my health
3. Actively managing my health
4. Social support for health
5. Appraisal of health information
6. Ability to actively engage with healthcare providers
7. Navigating the healthcare system
8. Ability to find good health information
9. Understand health information well enough to know what to do.

Each of the dimensions comprise four to six items and thus a total of 44 items.

Health literacy levels are assessed based on responses to the 44-item questionnaire with response categories ‘strongly disagree, disagree, agree, and strongly agree’ in scales 1 to 5 (part 1); and ‘cannot do, very difficult, difficult, easy, and very easy’ in scales 6 to 9 (part 2) [26]. High scores mean the respondents agree or find tasks easy, reflecting high health literacy; low scores mean that the respondents disagree or find task difficult, reflecting low health literacy [26]. The average mean score for the scales was used to select the dimensions of interest for the intervention. There was no specific cut-off score to select a scale, however, the scale with the lowest average mean score in each part of the questionnaire (Parts 1 and 2) were selected. A third scale was subsequently added based on the qualitative needs’ assessment.

Questions in the malaria questionnaire covered occurrence of malaria, knowledge on malaria.

including symptoms and treatment, treatment choices as well as associated malaria costs.

#### Qualitative needs’ assessment

In addition to the baseline survey, we used different qualitative data collection methods to gather data from different stakeholders with focus on management of malaria in the communities and its possible influence on health literacy. The design for the qualitative data collection follows an exploratory approach where different interest groups were identified to get a better understanding of community malaria programmes; a prospective programme base for the development of the health literacy intervention through stakeholder consultation, focus group discussions and individual interviews.

##### Participants

The research team suggested respondent groups of interest and finalized it in consultation with the District Health Directorate. The health administrators of the Ejisu Health Directorate served as entry points in the sampling process because they had first-hand information on the current state of malaria programmes and had easy access to the communities. Data sampling had a snow-balling effect as the health administrators reached out to other community gate keepers to identify 5 individuals for each group of respondents who could be enrolled into the study. Thus, in total the Health Directorate provided a list of eighteen [18] identified participants:

- five mothers with children under five years
- five community-based agents (CBAs)
- five community health nurses (CHNs)
- three health administrators (director, disease control officer, and health promotion officer)

Apart from participants from the Health Directorate, the others were representatives of.

the five sub-districts under the administration of the Health Directorate to get different perceptions from each sub-district.

##### Stakeholder consultation

After the baseline survey, we organised a stakeholder meeting for the heads of the health directorate, mothers, community health nurses and community-based agents. This was to get the diverse perceptions of stakeholders on existing community malaria management programmes, the challenges, and what could be done. We presented and discussed the findings concerning identified health literacy gaps and their relevance in developing an intervention. All stakeholders were given equal opportunity to express their opinion during this meeting. Information gathered were compared and analyzed together with the responses from the focus group discussions for each group.

##### Focus group discussions

To assess the perceptions of key stakeholders on malaria management in the communities, primarily community case management of malaria, we organised three focus group discussions for different five-member groups of key stakeholders: mothers, community health nurses and community-based agents. The focus group discussions [27] were moderated by research assistants to ensure that the five topics (Table 1) in the interview guide were discussed. The guide was developed taking into consideration the research question and the three key areas of health literacy; access, understand and application of health information.

**Table 1:** Topics for focus group discussions

1. Knowledge of community-based malaria programmes for children under five years	
2. Experiences with managing malaria through the community-based programmes	
3. Perceived benefits of the programme in managing malaria	
4. Perceived challenges of the programme in managing malaria	
5. Perception of the community-based programme on:	
a. Access to health information	
b. Understanding of health information	
c. Application of health information	

We analysed the responses from each group to understand the perception on the status of malaria management for children under 5 years and the health literacy potential of the existing programmes. Each group discussion lasted 1 h. The responses were recorded, transcribed verbatim and analysed thematically.

##### Individual interviews

Three individual interviews were carried out to gain knowledge in managing community-based programmes on malaria for children under five. The health administrators interviewed included the health director, the disease control officer and the health promotion officer. The interview with the director was conducted to gain insight into existing malaria programmes and the challenges related to community cooperation. From the interview with the disease control officer, our interest was to get insight into the existing strategies to control malaria in the community. The interview with the health promotion officer helped us know how the community promotes early treatment and preventive measures on malaria. The interview guide included questions on strengths and limitations of existing malaria programmes in the communities according to the different health administrators. These interviews also lasted one hour each. The responses were recorded and transcribed verbatim for analysis.

##### Observations at the child welfare clinics

Several recommendations from the qualitative data pointed to the use of the child welfare clinics as appropriate settings for an intervention; thus, we conducted observations at three of such facilities. Child welfare clinics are community-based health facilities set up to monitor growth and development of children under five years [28]. These clinics are run by community health nurses and community- based agents (CBA), who are volunteers, trained in management of childhood illnesses including malaria. Caregivers utilize these clinics every month to assess the health and development of their children.

The aim of the observations was to know how activities are carried out at the clinics, to identify gaps in the services provided at the clinic and how to integrate our intervention into current practices. The observation guide was based on the UNICEF recommended checklist [29, 30], which outlines steps on how a community child growth promoter or health nurse should provide positive counselling.

Two of the researchers carried out these observations in three child welfare clinics. In addition to the field notes, the use of the UNICEF checklist served as additional documentation of the observations. Observations were focused on the services provided at the clinic and not specifically the mothers. The three clinics were observed over a three-day period with one visit per clinic. The observations lasted for more than an hour for each facility and we observed how the mothers received services at the clinic from arrival to departure.

Furthermore, the analysis from our focus group discussions pointed towards the need for an interactive intervention approach. Further discussions among researchers led to the idea of game boards and leaflets as possible interactive materials, which was followed up by literature search on game boards in healthcare. Researchers identified the snake and ladders game board as a possible option based on the context of its use in other studies. After the observations, we therefore conducted informal interviews with some of the mothers where every other mother on completion of their visit was approached for an interview but only those who agreed were interviewed. The interview regarded the age of the child, the services mothers received at the clinic following the UNICEF checklist and their opinions on health information presented in the form of snake and ladder board game.

Fieldnotes were taken during both observations and interviews, summarized and analysed after the visits.

### Phase 2 co-production of materials

The second phase involved design of the intervention suitable for the subject of interest and the target population in consultation with stakeholders. The key stakeholders at this stage comprised all researchers (public health experts and communication designer), Ghana Health Service directorate staff (district directorate and health resource and learning materials unit), and community health nurses. Depending on the topic of discussion, each stakeholder consultation included the relevant stakeholders:

#### Game (co)-design

In developing the board game, discussion on the rules, manual and the design of the board game took place between the communication design expert and the other researchers. An iterative approach to design was used in line with the principles of co-designing. As such, the activities consisted of a non-linear process to define the problem and develop a solution. This involved formative evaluation throughout the processes of the design as well as a summative evaluation during testing of the game intervention by stakeholders. Based on the feedback from consultations with the district health director, the researchers continued to brainstorm to generate ideas on the design of the entire intervention. This involved an interactive discussion of ideas and creation of mind maps and further visualising these into concepts. Initial simulations of three models of the gameboard involved the designer and researchers to review design considerations to refine the objectives of the game design. This was followed by individual and group discussions with some mothers on their experiences with the gameboard and game play. The simulations and inputs from mothers bothered on layout, size of the gameboard, aesthetics and legibility of textual elements. The final versions of the board game were designed using a vector graphics software to ensure high-quality output and the ability to make changes after feedback from stakeholders.

#### Brochure co-design

The decision to cover the three themes on malaria, breastfeeding and referral system in brochures was based on the needs assessment. With respect to the brochure design, the researchers worked closely with the Health Resource and Learning Unit of the Ghana Health Service (GHS), a unit in charge of developing all materials on health education for the GHS. The malaria brochure was designed from scratch whereas the content of the breastfeeding leaflet was based on the UNICEF brochure on breastfeeding and complementary feeding [31]. Prior to collaborating with Ghana Health Service, the researchers deliberated on the layout of the brochures. We agreed that the brochures should be appealing to mothers with simple and precise messages and stimulating interaction among people. This decision was based on recommendations in other studies on design of health education and promotion brochures [32, 33].

Several drafts of the board game and brochures were discussed among the researchers before presented to other stakeholders. Thus, drafts of the intervention package were presented to the District Health Directorate and community health nurses for discussions on content of the board game and brochures. We sought their opinion on feasibility of using the tools considering the target group and the clinic setting .

### Phase 3- prototyping

The third phase involved testing of the materials for the intervention, intervention roll out and evaluation of fidelity. Here, the malaria health literacy game (Mosquito and ladders) in addition to the supporting brochures on malaria, breastfeeding and referral system were finalised. In August 2018, the researchers and key stakeholders met to go through the intervention and the roll out. The participants at the meeting were community health nurses from the selected communities, researchers, research assistants and representatives from the Health Directorates. The meeting served as both an introduction to the intervention as well as training of key facilitators of the intervention. The community health nurses facilitated the six- month intervention roll out by adding on the game play and use of brochures during health talks as a part of the activities at the child welfare clinics. Research assistants were trained at this stage to be observers of the intervention using a checklist with key features developed by the research team. This was to assess how much of the intervention was completed as planned. They worked together with the community health nurses to plan the schedule and to coordinate the logistical materials (brochures, and game boards and accessories).

### Stakeholders’ reflections post intervention

Stakeholders were interviewed during and after the intervention to reflect on the development of the intervention, its feasibility and sustainability. During the intervention delivery, 404 mothers, basically those who played the game (4 mothers per game for 101 clinic visits), were asked to comment on both the game play and the discussions based on the brochures. Post intervention delivery, a representative from the District Health Directorate, the community health nurses and the research assistants, respectively were interviewed online (via Zoom) as they reflected on the entire development and delivery of the intervention. Zoom interviews were conducted by a researcher outside of this study team to avoid bias in response due to familiarity. These interviews were transcribed with the interpretation presented in themes.

**Fig. 1:**
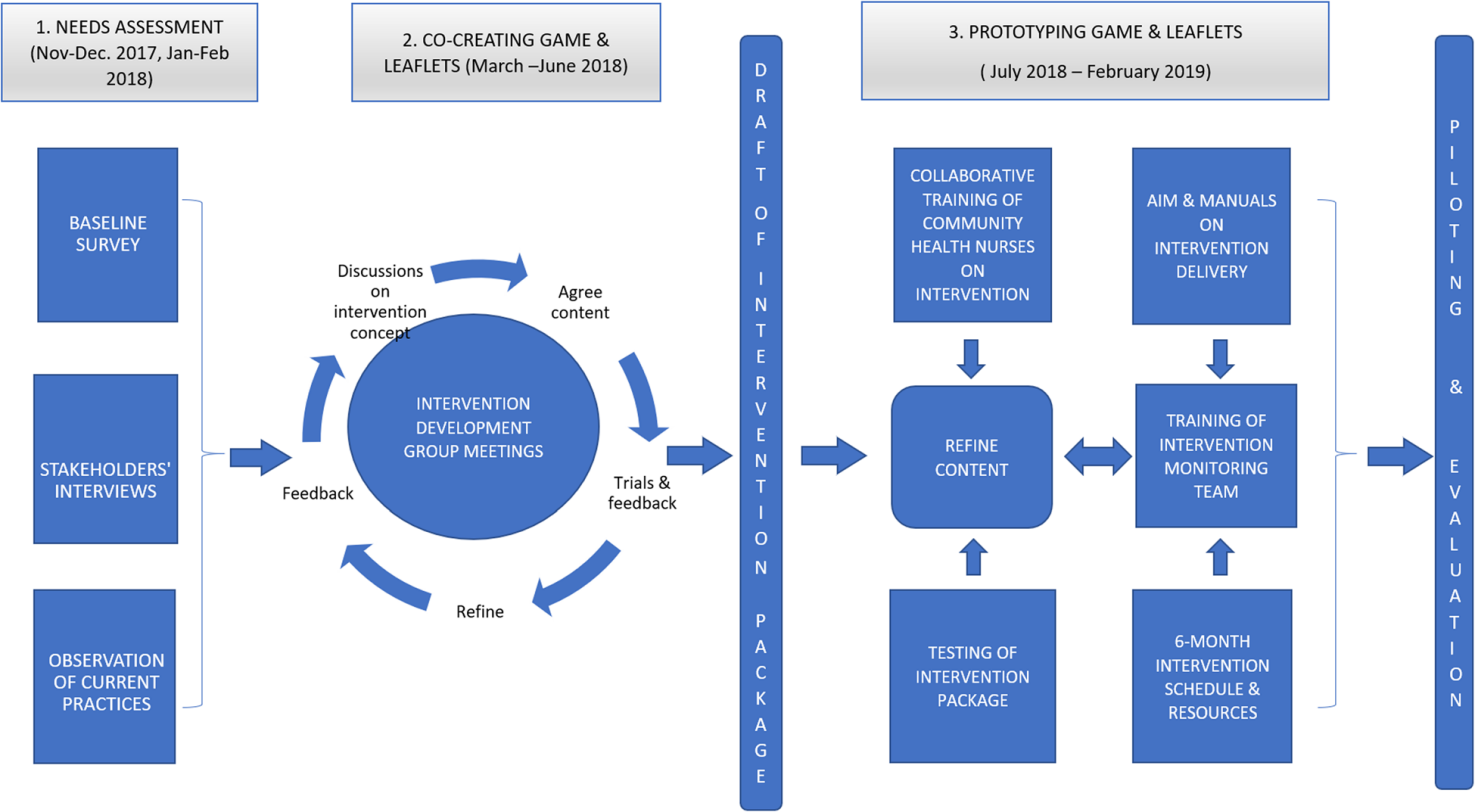
Conceptual Framework of Intervention Design

To summarise, the entire process has been captured in a framework as shown in Fig. 1, which includes the activities carried out at each stage of the development. The entire period from co-creation to prototyping lasted 16 months (November 2017–February 2019), with the pilot covering a six-month period from August 2018 to February 2019 in the 10 selected communities. To add on the reporting quality, the Guidance for Reporting Involvement of Patients and the Public (GRIPP2) checklist [34] is attached for transparency in accordance to International patients and public involvement study standards.

## Results

This section includes a descriptive summary on the baseline survey and the qualitative study (focus group discussions and individual interviews), a summarised table of results for the three phases in the development of the intervention and an elaborate presentation of the findings from the fidelity data and reflections from stakeholders.

### Phase 1- needs assessment

#### Quantitative assessment- baseline survey

Table 2 presents demographic characteristics of respondents. Most of respondents were female (98%), and the majority ranged between 20 and 39 years of age (85%). More than half of respondents (72%) had little to no education and a little over half (54%) were employed. Almost a quarter (38%) reported that they had some kind of illness or long-term disability.

**Table 2:** Background characteristics of survey respondents (*N* = 1234)

CHARACTERISTICS		
	EJISU-JUABEN (*N* = 631)	KWABRE (*N* = 598)
AGE ^**a**^		
15–19	4.1	3.7
20–29	39.8	46.3
30–39	42.8	40.6
40–49	11.2	8.5
50–59	1.1	0.5
60–69	1.0	0.3
SAMPLE	EJISU-JUABEN (635)	KWABRE (599)
EDUCATION		
≤ 9 years	65.8	79.6
12 years	20.7	19.0
≥ 12 years	3.5	1.4
EMPLOYMENT		
Employed	56.6	52.4
Unemployed	39.8	41.1
Retired	3.6	6.5
GENDER		
Female	97.3	97.8
Male	2.7	2.2
LIVING ALONE		
Yes	29.8	20.0
No	70.2	80.0
LONG TERM ILLNESS		
Chronic	9.6	9.0
Non-chronic	34.5	22.5
None	55.9	68.5
LANGUAGE		
Local language	90.1	88.7
English	9.9	11.3

**Note**^**a**^ **Five respondents were excluded (4 in Ejisu-Juaben; 1 in Kwabre), because the reported ages were invalid**

The survey helped to identify the health literacy scales where caregivers recorded low scores on the average and as well, outlines caregivers’ health seeking practices on an episode of malaria in children as shown in Table 3.

**Table 3:** Main findings from the baseline survey

Data source	Needs Assessment Baseline Survey
Objective	To assess health literacy needs and challenges in malaria management in the community
Stakeholders	• Caregivers with children under 5 years
	• Research assistants
	• Researchers
Results	• Out of the nine dimensions, two scales were selected based on the average mean score. Scale 1 Having Health Provider Support (2.44) and Scale 7 Navigating Health System (3.33)
	• On the management of malaria, 96% of caregivers had knowledge on the main symptom of malaria (fever); 23%; 47% of caregivers had children with recent episode of malaria and their treatment sources included 69% self-treatment; 31% hospital treatment and 0.3% did nothing as their first response to the disease. Most important challenge identified was seeking the right treatment source.

Detailed information on all scales is reported elsewhere

Thus, based on the survey, scales 1 and 7 were targeted for improvement in the intervention concerning health literacy needs of caregivers.

With the malaria questionnaire, we found that caregivers had high knowledge of symptoms of malaria; however, the majority resorted to self-treatment rather than consulting health facilities. Self-treatment covers using medication from home, buying medicine over the counter and using traditional medicine. Thus, caregivers had high knowledge of symptoms of malaria, which did not correspond to them seeking treatment from health facilities in line with Ghana Health Service recommendations.

### Qualitative needs assessment

#### Stakeholder discussion on survey results

During this discussion, we identified the child welfare clinics as a suitable setting for the intervention. As stated by one community health nurse:

*“I think if we include the programme and volunteers on the Community Health Nurses weighing sessions and reach out to and educate mothers on the programme it will be successful.” (Community health nurse)*

#### Focus group discussions

The mothers in this study were petty farm product traders aged between 20 and 45 years with different educational backgrounds from little or no education to tertiary-level education. Four [4] mothers were married and one was divorced but co-habiting with a new partner; the number of children for each woman ranged between 1 and 4.

The community health nurses included four [4] females and one male with an age range between 28 and 36 years. Seniority among community health nurses ranged between three [3] and nine [9] years.

The community-based agents in the study had other occupations: three were farmers, one was a teacher and one was a trader. The age range for the community-based agents was wide, 30–72 years, and out of the five [5], three [3] were females and two males.

In the analysis, five themes emerged, see Table 4. Caregivers’ patronage of malaria programmes could be attributed to the education content (theme 1) and the incentives given in the form of nudging mechanisms to improve early treatment and prevention of malaria (theme 2). In addition, some individuals and mother group agencies promoted preventive practices in the communities by initiating healthy habits to prevent malaria (theme 3). Agencies here, refer to individuals or group of mothers who act independently to promote healthy practices in the community. However, there were challenges with better understanding of health education because the content was presented in a one-way didactic approach (theme 4). This led to the inclusion of the health literacy dimension “Understanding of health information well enough to know what to do” (Scale 9 of the health literacy questionnaire) as target for improvement. Together with possible cultural barriers (theme 5), these last 2 factors (themes) challenged the success of the community programmes.

**Table 4:** Results from Focus Group Discussion with caregivers

Data source	Focus group discussion
Objective	To assess perceptions on community case management of malaria with focus on health literacy
Stakeholders	• Mothers
	• Community-based agents
	• Community health workers
Results	Perceptions on existing malaria programmes were grouped into five themes:
	• Mothers valued the programmes due to possibility of education; “*I was involved in the malaria programme because of my work as a prophetess (a religious female leader). People in the community do come to me with all kinds of sickness, so I thought it wise to involve myself to get the chance to be educated and to educate other people in the community in sleeping in the mosquito nets*”.
	• Nudging and reaching out through existing social platforms to promote healthy practices; “*I’ve heard about the malaria programme because even last year, they came to share mosquito nets to us*”.
	• Health education presented as instruction; “*Some of the mothers did not follow the instructions that was giving to us. They were supposed to dry the net in the sun before sleeping in but some of the mothers didn’t follow the instruction and there were complaints of facial itching*.”
	• Strong agency of mothers willing to support peers to understand and use health information; “*I and other mothers were also doing the sweeping of the venue every morning and evening to enhance or facilitate the sharing of the mosquito nets. We also saw it wise to come together to educate ourselves and other mothers in the community to prevent malaria*”.
	• Possible cultural barriers for health promotion.
	• Conclusion: A future intervention should focus on interactive health education during social gathering like the child welfare clinic; and inclusion of community health volunteers with incentives.

From the perspective of the health providers including the health volunteers and community health nurses, patronage of malaria programmes could be linked to easy accessibility of healthcare with no costs to caregivers and to the good relationship between volunteers and caregivers. The main challenge identified by providers was logistical problems in terms of medication and test kits, which resulted in the halt of the programme.

#### Individual interviews

All three administrators: the health director, the disease control officer and the health promotion officer, had long experience in their functions, with at least eight years of experience in health systems management [8]. The director and health promotion officer were both women. Table 5 shows results from the interviews.

**Table 5:** Results from Individual Interviews

Data source	Individual interviews
Objective	• To gain insight on existing malaria community programmes, challenges and strengths
Stakeholders	• District health promotion officer
	• District disease control officer
	• District health director
Results	The health administrators shared that:
	• the community case malaria programme has been put on hold for the past 2 years due to logistics on medical supply; “they brought the medicine for them to use and after the medicine was finished, that was the end of the programme, they did not receive any thing for the treatment, the challenges had to deal with the logistics that were never supplied.” Health administrator1
	• Social gatherings like churches or child welfare clinics appropriate for malaria programmes; “I think for a health literacy programme, we have to use the community health nurses, because you know the mothers will bring their children for weigh-in if nothing at all, for immunization.” Health administrator 2
	• Non-functional but existing mother support groups
	• Possible use of social media applications for health education.

The individual interviews with the health administrators showed that the community malaria programme, which was of primary interest in this study, had been halted in the communities but that programmes on malaria still ran at the child welfare clinics (CWC). Administrators recommended the use of social gatherings such as churches or child welfare clinics with community health nurses as possible settings for intervention on health literacy for caregivers.

#### Observations at child welfare clinics

The findings from the survey and focus group discussion suggested the importance of the social nature of the communities and thus, the need for moving from an instructional to an interactive approach.

Through the observations from three clinics, we mapped out which and how services were delivered at the CWC. Here, we considered when to possibly include the intervention and sought some perceptions on the concept of the game as a health education tool. The observations at the three clinics visited showed service delivery in accordance with the UNICEF checklist. Activities included health talks, weighing of babies and infants, immunization, and advice on child growth. They were all facilitated by two community health nurses and assisted by at least one community health volunteer. Being a religious society, all clinics started with a prayer and then a health talk; however, caregivers had a long waiting time for their turn to do the other activities after the health talk. This long waiting time was considered suitable for the health education sessions using the game. Caregivers knew of the suggested game board (Snake and Ladders) and perceived the idea as interesting and worth experiencing.

In summary, the baseline survey, together with the focus group discussions, interviews, observations and discussions identified the need to design an interactive health literacy intervention for caregivers to address three dimensions: having health provider support, navigating the health system and understanding health information. The child welfare clinics were recommended as suitable setting for this intervention.

### Phase 2 co-creation

#### Mosquitoes and ladders game

The snake and ladder board game was adapted and converted into the Mosquitoes and ladders game, to be used as an educational tool on malaria at the child welfare clinic. Considering the low literacy levels of the population of interest in the study setting in addition to the interactive aim of the intervention, this game was considered appropriate.

The collaborations with the communication design researcher on ideation and mind maps yielded a draft layout, colour schemes, graphic elements, rules for the game play and identification of the right materials to develop the board game. Revisions from simulations included addition of illustrations on the game board and strategic allocation of mosquitoes on the game with at most four mosquitoes on each row of the ten boxes.

**Fig. 2:**
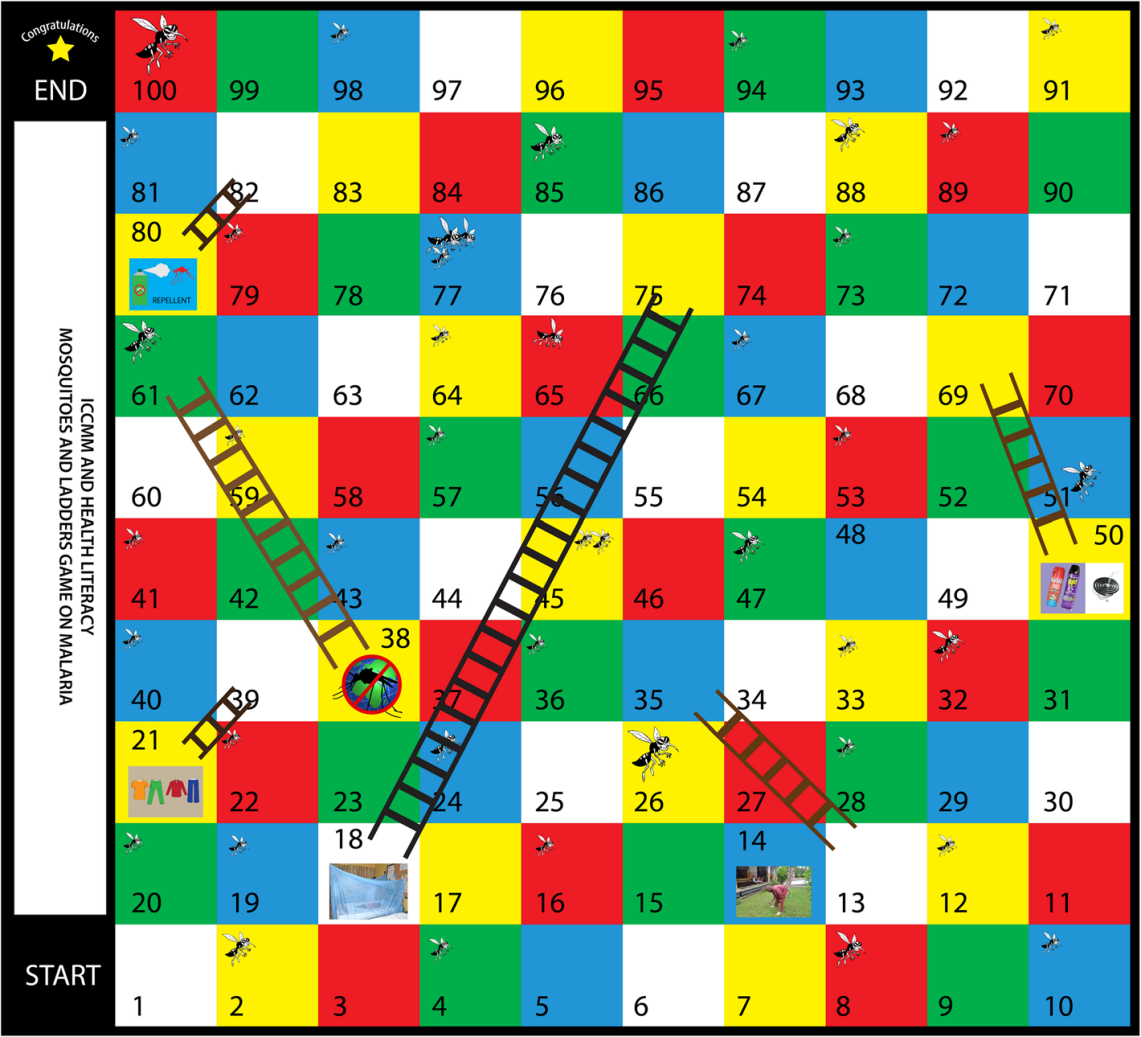
The Mosquitoes and ladders game

The iterative process of design resulted in a flat panel game in a ten by ten grid of five colours, red, yellow, green, blue and white as shown in Fig. 2 below. The questions for game play were matched with four of the colours (except white) on the board game and categorized into four themes: cause (red), symptoms treatment (green) and prevention of malaria (blue).

In addition to the design of the board game, the researchers (health and communication design researchers) put together the intervention manual, the rules, questions and answers to be used in the game play. Overall, the revisions and discussions improved functionality and understanding of the game design by users.

#### Brochure co-design

Three brochures were developed in addition to the game for use as health education tools covering malaria, child nutrition (breastfeeding) and referral system in the Ejisu-Juaben district health directorate. The additional brochure on breastfeeding emerged during the focus group discussion with caregivers, as an important topic of need and, that on referral system was to address scale 7 on navigation of the health system.

The three brochures were co-designed with precise messages and clear pictures to enhance interaction and understanding of the messages as intended. The first pages were carefully designed with little text and a picture that would appeal to users. Each brochure was designed with an A4 sheet and the content of the malaria and nutrition brochures covered a front and back of the sheet and made into four folds. However, the referral brochure covered one page as it mapped out the referral chart of the district. Based on comments from other stakeholders, the brochures were reviewed with much interest in concise messages with well informing pictures. Revisions were made until reaching the final prototypes.

### Phase 3 prototyping of game and brochures/ intervention assessments

Trials of the game and brochures resulted in changes in some of the questions and responses for the game for better clarity. The process showed the need to translate the questions and responses of the game into the common local language, Akan-Asante Twi.

In the six-month pilot stage period from August 2018 to February 2019, the community health nurses facilitated the game play among the mothers or caregivers while they used the brochures as tools for health education as a part of the services at the clinics. This was conducted in 10 selected communities.

### Fidelity assessment

Fidelity with the intervention design in the implementation was assessed through observations of the intervention during the six-month pilot phase showed a high participation rate. The response to the observation checklist was a “yes” or “no” and in line with these responses, almost all the steps outlined in the manual were duly followed (Table 6).

**Table 6:** Intervention Fidelity Assessment: Compliance with intervention implementation design (*N* = 101 clinic visits)

Item	Intervention Fidelity Assessment (101 respondents)	Yes %	No %
1	Did community health nurses give a recap of previous months’ sessions?	85.1	14.9
2	Was the first hour of the day’s session covered by brochure discussions and game play?	83.2	16.8
3	Was the brochure discussion held before the game discussion?	91.1	8.9
4	Did all caregivers present have brochures for discussions?	87.1	12.9
5	Did the Community Health Nurse engage mothers during the discussions?	93.1	6.9
6	Did caregivers participate in the brochure and game discussions?	94.1	5.9
7	Were caregivers given time for questions with feedback?	92.1	7.9
8	Was the game played in groups?	87.1	12.9
9	Did community health nurses explain the rules of the game to caregivers?	86.1	13.9
10	Did the community health nurses facilitate the game discussions?	81.2	18.8
11	Did the game lead to further interactions with or questions to community health nurse?	86.1	13.9
12	Did the community health nurse use the platform to elaborate further on the questions and answers to the caregivers?	86.1	13.9
13	Were caregivers interested in the game?	86.1	13.9
14	Did the game promote group interaction?	89.1	10.9
15	Did the community health nurses refer to illustrations and pictures in the brochure in the discussions?	79.2	20.8
16	Did the facilitator introduce the topic for the next discussions session to caregivers?	82.2	17.8

The least observed activity was on nurses referring to the brochure illustrations, where only 79% of observations were confirmed out of 101 clinic visits. Items 6, 11 and 14 were selected to assess the level of interaction of the intervention and on the average, the intervention was observed to be 90% interactive.

#### Stakeholder reflections on intervention development

The following summary describes reflections by some stakeholders based on their involvement in the design and roll out of the intervention in the pilot phase. These interviews were pre-coded with the themes on the advantages of adapting co-creation in the design of the intervention. The summary here does not touch on effectiveness as it did not result in any quantified outcomes. The themes included usefulness, ownership and sustainability of the intervention.

##### Usefulness of the intervention

In summary, the stakeholders’ reflections showed that the relevance of the intervention lies in the local needs driven basis, which resulted in the production of useful health education materials in terms of both content and delivery approach. The intervention promoted better understanding of health information given the illustrative pictures on the game board and in the brochures as shown in the quote:

*“I have learned a lot about the things to do to prevent malaria; like the man weeding on the ludu (game) which tells me that when I weed around my house it can prevent mosquitoes from breeding”. Mother*

Again, the interactive approach to health education promoted health provider- caregiver relationship as was expressed by one community health nurse:

*“We need to interact with them, consult them and know, what is happening with them, because through this some of them were able to come out with what their problems are. Initially they would think that the nurse is only interested in weighing my child and know how heavy he or she is, and that is it. But now we are teaching something different so when the person has a problem, she can come to you oh madam, this and that is what is happening to me and so what could I do? And then the advice that you have you can give to the person.” Nurse*

In terms of health provider-caregiver relationship, the study aim was to enable caregivers to easily approach healthcare providers with health concerns and as was experienced during the intervention pilot, both healthcare providers (nurses) and caregivers saw the essence of engaging with each other for better healthcare.

##### Ownership

An advantage of co-creating interventions with users of the intervention is the sense of user ownership. In this study, the degree of a sense of ownership was expressed differently by stakeholders because the study introduced stakeholders in different phases of the development based on the objective of the phase. Thus, while some stakeholders like the District Health Directorate were engaged from the beginning of the development of the needs’ assessment through to the pilot phase, others like the community health nurses were introduced during the feasibility testing of the materials produced. Although representatives of the end users including caregivers and nurses were involved in the early stages, they were not automatically the end users: thus, the sense of ownership for the end users was expressed differently than expected.

##### Sustainability

In co-creation, the sense of ownership promotes possibilities for long-term sustainability of interventions. In this study, the sense of ownership, especially by the directorate, and usefulness of the intervention to end users accounted for the sustainability strength of the intervention. However, this was challenged by availability of resources and top administrative buy-in. Therefore, although the district health directorate with its current leadership, plans to review the use of these materials and its delivery approach as part of the health education approach in the district, sustainability is challenged by high attrition rates of community health nurses, a problem deeply rooted in the Ghanaian health system. The stakeholders expressed this problem in the quotes below:

*“What we are experiencing now is a high attrition rate. If a community health nurse goes into midwifery and is no more at the service area for child welfare, this means we lose, especially when the knowledge is not passed on”. Health directorate*

*“I used to go to the outreach facility but now I don’t go because I have taken over as an in-charge (higher position). The person who took over was in school and had just graduated so she has not started work yet. The one who filled in for her was also temporary, so I didn’t introduce it to her”. Nurse*

Stakeholders recommended training of all community health nurses using these health education materials to ensure continuity in the event of community health nurses reshuffling. Again, one stakeholder suggested inclusion and review of the interactive approach as one of the recognized health education approaches in the district to promote sustainability of the intervention.

*“So, during our reviews in fact, it’s an area we will review. And once it’s something that we are reviewing, it means it’s on our radar as one of the methods of service provision. Then we need to put in more effort to scale it up, otherwise, your guess is as good as mine”. Health directorate.*

In summary, the sustainability strength of this intervention lies in its usefulness to users (nurses and caregivers) but its weakness lies in availability of resources and administrative buy-in.

## Discussion

This paper describes the development of a community-based intervention focusing on health literacy to increase knowledge on prevention and early malaria treatment practices among mothers with children under five years in Ghana.

Co-creation is a collaborative approach to develop public health interventions including both academics and stakeholders (non-academics) [11, 15]. Unlike traditional top-down approaches to intervention development [35], co-creation leads to tailored interventions which address locally identified problems with local solutions [11, 35]. The needs assessment phase in this study outlined gaps in the health literacy levels of caregivers as well as gaps in the management of malaria in the communities for children under five years. These findings resulted in the design of an interactive approach to health education through development of a game and brochures to support discussions. Thus, the content of the materials developed were practically useful to caregivers and the delivery of the intervention fitted the setting of child welfare clinics. Hawkins et al. [16] pointed out that the participation of stakeholders in the co-creation of intervention content provides a way to tailor intervention content to the context and target population, thus maximising acceptability and reducing challenges concerning implementation [16]. This study achieved a high patronage with almost 90% of caregivers showing interest through high participation in this interactive approach to health education. The high patronage could be attributed to the setting (Child Welfare Clinics). The acceptance of the game and brochures can be ascribed to their usefulness in meeting the knowledge gap in the appropriate management of malaria.

In addition to content relevance, the layout and design of the game and brochures with precise messages facilitated understanding of the message, which was part of the aim for developing the materials. In the development of health education materials the content, layout, text construction and lexical comprehension impacted on the legibility [33]. A study by Michel et al. in the United Kingdom showed that parents’ understanding of information influenced child care [36, 37]. In their study, parents who understood the meaning of ‘wheezing’ were more likely to report ‘wheezing’ in their children [36]. The use of simple messages, pictorial layout of the materials as well as translation of some of the messages in the local language in this study, enhanced the understanding and relevance of the materials developed.

The use of these interactive materials also influenced perceptions on the roles of a health provider in health care delivery. The use of interactions in health education is a way to confirm comprehension of information and it improves provider-patient communication [37]. In addition, low patient oral and aural literacy is associated with poor health outcomes. Thus, Nouri et al. stipulated that oral exchange is relevant to improve health provider relationship and health outcomes [38]. Health provider support was one of the dimensions in health literacy identified as the primary concern in terms of health literacy. Thus, the aim of the interactive materials was to break barriers of communication and improve the relationship between the health provider and caregivers. As shared in the reflections, caregivers after such interactive sessions reached out to nurses to share other health concerns and sought advice. In other words, the social nature of the intervention, which is opposite to the previous one-way didactic approach to health education, broke barriers in the communication between nurses and caregivers.

Ownership is a concept within co-creation, a state, right or act of possessing something [11, 16, 39]. The varying range of possible engagements with different kinds of stakeholders [11] yields varying degrees of ownership in the co-created intervention. This study engaged the different stakeholders based on the aim at each phase and activity in the development process. While stakeholders from the health directorate were involved during the entire study, other stakeholders were consulted when necessary especially during the development of the materials; for some stakeholders like mothers, different mothers were consulted at different times. Therefore, although stakeholders including mothers shared a sense of ownership of the intervention, the degree of the sense of ownership was stronger for those who were more engaged. This is possible in co-creation as O’Cathain et al. [10] shared that the principles of when, where and how to engage stakeholders in co-creation is still an open question to be addressed. Ownership is said to promote adherence and results in sustainable interventions, and the high participation and interest that users showed in this study promotes sustainability.

The sustainability strengths of this intervention lie in the interest of users and the individual stakeholders, but leaves more room for long term sustainability at health system level. Unfortunately, the challenges in the health system in this context also challenges the sustainability of this intervention. Specifically, issues of high attrition rate and regular reposting of nurses challenge the sustainability of this intervention. The high attrition rates of community health workers in most developing countries is well documented [40, 41]. Abbey et al. [42], reported an attrition rate of 22% in a 30-day period for community health nurses. This relatively high rate was associated with influence by immediate family, remuneration, and other job opportunities [42]. However, training of more health workers and Ghana Health Service’s approval of these interactive materials as recommended tools for health education in the communities, could address the limitations on sustainability of this intervention resulting from high attrition rates. This study recommends that, if possible, other community health workers like the well-known community-based agents (volunteers) should be trained to use these materials to educate mothers in the community.

### Strengths

We adapted this pragmatic approach to reporting on the intervention as it makes room for reporting on the development of the materials and steps of the intervention, which is sometimes difficult to capture using other guidelines [6, 43–45]. This study recommends this approach as useful in reporting on interventions in relation to both process and content.

To our knowledge, this is the first study to adopt the combination of a game and brochures as useful health education tools for caregivers to promote understanding of health information and health provider support. Considering the social nature of the context, the study used the strengths of the society to meet the needs and makes it possible for other researchers to explore how to best use this principle to address gaps in the society.

The use of interdisciplinary researchers in the development of the game and brochures improved the usefulness of the layout and design of the materials as well as provided better solutions to societal problems.

### Limitations

From the translated version of the HLQ, one of the identified health literacy scales did not seem to fit well in the confirmatory factor analysis [25]. Specifically, in the factor analysis, the construct ‘understanding health information’ did not seem to measure what it was intended to measure which influences its interpretation [25]. However, in this study, the qualitative findings also influenced the selection of the scales and this showed that understanding health information was a relevant dimension for the intervention.

The use of co-creation and a sense of ownership sometimes becomes a disadvantage. As noted from the table on the fidelity assessments, none of the planned activities was 100% observed during the intervention delivery. Thus, for each activity planned, community health workers deviated from the required process of delivery, showing that the concept of co-creation was still in use in the pilot phase. However, this might also be observed during implementation after piloting probably to suit current conditions; hence, it calls for monitoring to improve impact of the intervention.

Using an approach in which stakeholders were not always consulted together, we might have lost some good ideas as the deliberations might have yielded constructive suggestions. In addition, it would have been appropriate for all stakeholders to be involved from the onset to give each stakeholder an equal chance to contribute. Therefore, better approaches to obtain useful inputs from experts individually and as a team in the design of interventions could be useful.

A larger group size and more focus group discussions in this study may have enriched the data for the study, however, the consolidation of the data from interviews and the focus group discussions improved the usefulness of the intervention. Though the focus group discussion of the caregivers included only five respondents, the analysis of the data provided an in-depth understanding of the caregivers and gave us a good idea of what caregivers need in a health literacy intervention on management of malaria in children under five.

## Conclusion

This study outlines the three stages involved in the development of a tailored health literacy intervention for mothers with children under five years in the management of malaria at community level in Ghana. The interactive nature of this approach to health delivery interventions led to better caregiver-health provider relationship and a sense of recognition of a more participatory approach to health delivery. A stronger buy-in at the top-level of health management and scaling it out to communities would improve sustainability and reach a larger audience.

## Supplementary Information

**Additional file 1.**

## Data Availability

The datasets used and analysed during the current study are available from the corresponding author upon reasonable request.

## Abbreviations

HLQ: Health literacy questionnaire
CHPS: Community-based health planning and service
CHN: Community health nurse
OPHELIA: Optimal health literacy
GHS: Ghana health service
UNICEF: United Nations children’s fund
CWC: Child welfare clinic
