# Supplementary material for: Co-creation and prototyping of an intervention focusing on health literacy in management of malaria at community-level in Ghana
Source: Res Involv Engagem. 2021 Aug 5;7:55. doi: 10.1186/s40900-021-00302-0 (PMC8340491; doi:10.1186/s40900-021-00302-0)
Supplement: Supplementary file 1 — Additional file 1. [file 40900_2021_302_MOESM1_ESM.docx]

# MANUAL FOR HEALTH LITERACY INTERVENTION IN EJISU JUABEN MUNICIPALITY

The basis of this manual is on a PhD study conducted by Millicent Addai Boateng on Assessment of the impact of ICCmM on management of malaria on health literacy, child morbidity and its associated cost. Faculties from Aarhus University, Denmark and Kwame Nkrumah University of Science and Technology, Ghana supervise the project. The objective of this study is to:

**To design a relevant community health literacy intervention and assess its usefulness with respect to health literacy of caregivers and child morbidity in the Ghanaian context**

We conducted a baseline survey as well as qualitative study as needs assessment and the findings served as the basis for the development of this health literacy intervention. Below is a description of the intervention, content and guidelines to assist in the appropriate execution of the intervention plan.

# Health Literacy Intervention.

Health Literacy has been defined by WHO as the cognitive and social skills which, determine the motivation and ability of individuals to gain access to, understand and use information in ways which promote and maintain good health. Health Literacy means more than being able to read pamphlets and successfully make appointments. By improving people's access to health information and their capacity to use it effectively, health literacy is critical to empowerment.

Based on this definition and findings, my study designed a game which is very common in the Ghanaian setting on malaria to improve access, understanding and easy application of malaria health information. Although the intervention has a lot of information to offer, the focus of the intervention is the mode of information delivery to promote health literacy of caregivers with children under 5 on management of malaria. The objective of the intervention is to:

- Engage caregivers in the active management of health concerns of their children under 5
- Promote access to malaria information
- Improve understanding of health information
- Promote appropriate navigation of the health system.

# INTERVENTION CONTENT

The intervention includes a game on malaria and brochures on malaria, nutrition and referral structure in the municipality of study. Since the study had malaria as the health problem of concern, the game was designed to cover this topic, but the other two topics of interest are highlighted topics of concern which came up during the baseline survey and qualitative study.

# The Game-Malaria and Ladders

This game was developed based on the original game, Snake and ladders, a very popular group game in Ghana. The original game is played by families, friends, colleagues and among children. This particular game concept was used due to the key focus of the intervention, to promote interactions during health education for better understanding and better involvement of caregivers to properly manage malaria in children and the family. The description and guide for the game is provided in addition to the game board.

# The Brochures

Three brochures have been designed on malaria, breastfeeding/complementary feeding and referral system in the Ejisu Juaben municipality. The brochures on breastfeeding and complementary feeding was extracted from brochures by UNICEF on the topics but that on malaria and referral system were designed with the support of the Health Learning Materials Centre under the Ghana Health Service. The brochures have been flooded with pictures for easy recognition and better understanding as has been shown in other studies.

Below is the schedule on the use of the intervention tools for the 6 months period

| **Month** | **Brochures** | **Game** |
| --- | --- | --- |
| 15th August- 14th September | Malaria | Played |
| 15th September- 14th October | Breastfeeding | Played |
| 15th October-14th November | Referral system | Played |
| 15th November-14th December | Malaria | Played |
| 15th December-14th January | Breastfeeding | Played |
| 15th January-14th February | Referral system | Played |

# Intervention Delivery Venue

The intervention will be rolled out in 10 communities in the Ejisu Juaben municipality in Ashanti region in Ghana. The venue for intervention delivery is the child welfare clinics in each community which, is held at least once a month and each caregiver has to attend once a month. This venue or meet up point was selected since it a common gathering point for all caregivers to weigh their children and take vaccinations as well. Each caregiver is supposed to attend the clinic till the child is 5 years and this covers the category of caregivers for the study. In attendance are caregivers, community-based agents (volunteers) and community health nurses (CHNs) who facilitate the weighing and vaccinations exercises. Prior to weighing and vaccinations, the CHNs provide health talk and this period will be used for the intervention delivery.

# Mode of Intervention Delivery

The health literacy programme will cover the first hour of the meeting. The first thirty (30) minutes will be an interaction on the brochure for the month which will be led by the CHNs. After the interaction, then four groups of mothers as outlined in the game guide will play the game. CHNs should involve mothers as much as possible in the brochure interaction session. **Both caregivers and CHNs will have a copy of the brochure during this session so CHNs should allow mothers give their views on what they see in the brochures based on the pictures before the CHN provide an elaborate explanation.** We hope to enhance mothers’ engagement and participation to improve understanding of the topic under discussion.

# Strategies To Check Caregivers’ Understanding

After the interaction, either CHNs should ask for feedback through questions or repetition of what was discussed by some mothers. On the next month’s meeting, CHNs should do well to ask of questions from the previous months’ discussions before taking up the topic for that month. Since the topics will be repeated in the last 3 months, CHNs should allow the caregivers to lead the interaction based on the same brochures and if necessary, the CHN should provide clarification when needed.

# Game Time

The game must be played six times or more (in the case of those CWC centres who have multiple meetings a month) in the six months period if possible but otherwise, at least 4 times in the six months’ period. CWC centres that meet more than once a month should repeat the game and interaction on every meeting since different mothers attend on every session.

The programmes organizers from the health directorate office will lead the interaction on the referral system.

# Programme Wrap Up

The six months programme will be crowned with a community durbar where 20 caregivers from the 10 communities will represent their communities in the final game play among communities. This programme will serve as a good gathering to create awareness of what has been done in the municipality for the past six months and assess how much caregivers learned from the game.
